# MFEAFN: Multi-scale feature enhanced adaptive fusion network for image semantic segmentation

**DOI:** 10.1371/journal.pone.0274249

**Published:** 2022-09-30

**Authors:** Shusheng Li, Liang Wan, Lu Tang, Zhining Zhang

**Affiliations:** State Key Laboratory of Public Big Data, College of Computer Science and Technology, Guizhou University,Guiyang,Guizhou,China; PDPM IIITDM: PDPM Indian Institute of Information Technology Design and Manufacturing Jabalpur, INDIA

## Abstract

Low-level features contain spatial detail information, and high-level features contain rich semantic information. Semantic segmentation research focuses on fully acquiring and effectively fusing spatial detail with semantic information. This paper proposes a multiscale feature-enhanced adaptive fusion network named MFEAFN to improve semantic segmentation performance. First, we designed a Double Spatial Pyramid Module named DSPM to extract more high-level semantic information. Second, we designed a Focusing Selective Fusion Module named FSFM to fuse different scales and levels of feature maps. Specifically, the feature maps are enhanced to adaptively fuse these features by generating attention weights through a spatial attention mechanism and a two-dimensional discrete cosine transform, respectively. To validate the effectiveness of FSFM, we designed different fusion modules for comparison and ablation experiments. MFEAFN achieved 82.64% and 78.46% mIoU on the PASCAL VOC2012 and Cityscapes datasets. In addition, our method has better segmentation results than state-of-the-art methods.

## Introduction

Semantic image segmentation aims to annotate each pixel in an image with semantic information, a challenging task in computer vision. Fully convolutional networks (FCNs) [1] are pioneering work in deep learning segmentation networks. The essential innovation is convolutional layers instead of fully connected layers, enabling end-to-end semantic segmentation by generalizing pixel points in an image with the same semantic meaning. However, FCN classifies each pixel without considering how they relate to each other.

To obtain more abundant context information, improve the accuracy of segmentation results. Researchers have proposed methods for aggregating contexts, such as pyramid pooling modules (PPM) [2] and atrous spatial pyramid pooling modules (ASPP) [3]. PPM [2] can aggregate multiscale contextual information to obtain global context. ASPP [3] uses dilation convolution to increase the receptive field size without adding additional parameters. However, using a single pyramid pooling module, the image contains objects of different sizes in the same class and cannot capture too large or too small targets well. Therefore, we designed a double-branch structured feature extraction module, Double Spatial Pyramid Module (DSPM), which consists of two parallel Spatial Pyramid Modules (SPM1 and SPM2) with different atrous rates. SPM1 is used to capture small objects in the image by using the dilation convolution with lower atrous rates, and SPM2 is used to capture large objects in the image by using the dilation convolution with more significant atrous rates.

The feature fusion is commonly performed by summation or concatenation operations, but this approach is ineffective. Because features at different levels or scales contain different semantic information. For features at different levels, lower-level features contain more location and detail information, while higher-level features have rich semantic information. Features at different levels contain different feature representations; simply using the summation or concatenation operation might consider the different information in each feature map equally, which could result in spatial and semantic information interfering. To more effectively fuse the features at different levels or scales, researchers have borrowed the idea of an attention mechanism in designing the network model. BiseNetV1 [4] proposed a feature fusion module (FFM) that concatenates the output features of spatial path and context path, generates channel weight vectors through a global pooling operation, and then reweights and fuses the features through two fully connected layers. LwMLA-NET [5] proposes the Multi-Level Attention (MLA) Module, which uses proposed spatial attention, channel attention, and pixel attention to extract relevant contextual information from different levels of abstraction, significantly reducing the computational cost by avoiding the propagation of unimportant features to the decoder. However, the attention blocks in MLA are connected in a serial configuration and do not enhance the adaptive fusion of these features as our proposed FSFM module can. Furthermore, MLA uses pooling. In contrast, FSFM uses a two-dimensional discrete cosine transform (2DDCT) to reduce the loss of information caused by pooling operations. Sknet [6] is an attention mechanism based on a convolution kernel. The feature maps obtained from split input feature maps are reweighted for adaptive fusion features. Sanghyun Woo et al. [7] proposed an attention mechanism module, CBAM, which combines channel and spatial to generate attention charts along spatial and channel dimensions for adaptive feature optimization. The above three methods apply the global average pooling (GAP) operation to obtain global information. However, FcaNet [8] demonstrated that it is challenging to obtain complex information about the input features by simply using GAP. Inspired by signal processing, FcaNet used the two-dimensional discrete cosine transform (2DDCT) [9] to transform the image to the frequency domain to obtain more frequency components, including GAP. We designed a Focusing Selective Fusion Module (FSFM) based on these observations. Specifically, the input FSFM feature map is first transformed through a spatial attention mask to obtain the weight vector of the spatial dimension; then, the feature map is transformed into the frequency domain by the 2DDCT transform to obtain more frequency components for enhancing the adaptive fusion features. Based on two essential modules, FSFM and DSPM, we propose a multiscale feature-enhanced adaptive fusion network named MFEAFN to improve semantic segmentation performance. In summary, our contributions are:

- We design a Focusing Selective Fusion Module (FSFM), which can enhance the adaptive fusion of these features by generating spatial and frequency correlation weight mappings for each feature map. FSFM is not only used to fuse features at different levels but also to fuse features with contextual and global information.
- A Double Spatial Pyramid Module (DSPM) was designed to extract objects of different sizes from the same category more efficiently.
- Based on the DSPM and FSFM Module, we designed a Multi-scale Feature Enhancement Adaptive Fusion Network named MFEAFN. We achieved 82.64% mIoU in PASCAL VOC 2012 and 78.46% mIoU in the Cityscapes datasets. Compared with the state-of-the-art methods, we have achieved better results.

## Related work

### Semantic segmentation

The FCN [1] provides a new way of exploring semantic segmentation by completing the recognition accuracy of images from image-level recognition to pixel-level semantic segmentation in a fully supervised learning approach to image semantic segmentation. Networks based on fully supervised learning image semantic segmentation methods can be divided into four major categories, namely, feature fusion-based methods (e.g., Pyramid Parsing Module [2] and attention modules [10]), probabilistic graph model-based methods (e.g., CRF [11] and MRF [12]), methods based on optimized convolutional structures (e.g., dilation convolution [13], hybrid dilation convolution [14] and Depthwise Separable Convolution [15]), and methods using encoder-decoder structure methods [16–20].

### Spatial pyramid pooling

Spatial pyramid pooling (SPP) [21] uses different window sizes and step sizes for different output scales to ensure that the output scales are the same and can fuse multiple-scale features to capture rich contextual information. ICNet [22] divides the images into high, medium, and low resolution layers. The low-resolution images are first allowed to pass through the semantic segmentation network to generate coarse segmentation results; afterward, the cascade label guidance and cascade label guidance strategy integrate the medium and high resolution features to optimize the previously generated coarse segmentation results progressively. PSPNet [2] improves the ability to obtain global information by aggregating contextual information from different regions through the pyramid pooling module. DeepLabv2 [23], DeepLabv3 [3], and DeepLabv3+ [18] apply several parallel atrous convolutions with different rates (called Atrous Spatial Pyramid Pooling, or ASPP) to capture rich contextual information. DSNet [24] proposes a Context-Guided Dynamic Sampling (CGDS) module that adaptively samples spatially useful segmentation information in spatial by obtaining an efficient representation of rich shape and scale information. APCNet [25] proposes the Adaptive Context Module (ACM), which uses the GLA to compute the context vector for each local location to aggregate contextual information. SpineNet-Seg [26] is a network discovered by NAS, scale-Perarm network semantic segmentation. CFPNet [27] proposes the Channel-wise Feature Pyramid (CFP), a module that jointly extracts feature maps of various sizes and reduces the number of parameters. FPANet [28] designed a lightweight Feature Pyramid Fusion Module (FPFM) to fuse two different levels of features.

### Attention module

The basic encoder-decoder encoder compresses the information of the whole sequence into a fixed-length vector, causing information loss. The attention mechanism was subsequently introduced in computer vision for target detection [29] and semantic segmentation [30]. The model structure of the soft attention mechanism is divided into three attention domains: the channel domain, the spatial domain, and the mixed domain. The signal on each channel is given a weight in the channel domain to signify the channel’s significance to the essential information. Typically, channel masks are created first, and then significance is assessed for each channel, representing SENet [31]. SENet first pooled the scaling factor globally for each channel to obtain a scalar called Squeeze. Then, the original channel element was multiplied by the corresponding channel’s weight to obtain a new feature map. Sknet [6] is an enhanced version of SENet, which can adaptively adjust its receptive field by using different weights of the convolution kernel for different images. Spatial domain key information is extracted by the spatial transformation of spatial domain information in images. Usually, a spatial mask of the same size as the feature map is formed, and then the importance of each position is calculated, representing the Spatial Attention Module. The attention of the spatial domain is to ignore the information in the channel domain and treat the features in each channel equally. This approach will limit the spatial domain transformation method to the feature extraction stage of the original image when applied to other layers of the neural network layer. The attention of the channel domain is to the global average pooling the information in a channel and ignoring the local information in each channel. The attention mechanism model of the mixed domain is designed. At the same time, the importance of channel and spatial attention is calculated, representing BAM [32] and CBAM [7]. DANet [30] introduces spatial and positional attention to resolve the differences between pixels of a category arising during the convolution process. EMANet [33] then proposes the Expectation-Maximization (EM) algorithm to learn attention features, solving problems such as the computationally intensive DANet.

### Discrete cosine transform

A discrete cosine transform is a standard tool in the field of signal processing. In recent years, several applications introducing discrete cosine transformation have emerged in computer vision with the development of deep learning. To classify images encoded by DCT, Ulicny et al. [34] used a CNN. By feeding the rearranged DCT coefficients to the CNN, Lo et al. [35] accomplished semantic segmentation on the DCT representation. FcaNet [8] was cut from the frequency domain, and the authors proved that the global average pooling (GAP) and the 2-D discrete cosine transform (2DDCT) lowest frequency component is proportional, and more frequency components are introduced through the DCT transform to utilize the information thoroughly. Shen [36] et al. proposed a new mask representation by applying the discrete cosine transform (DCT) to encode a high-resolution binary-valued grid mask as a compact vector.

### Backbone network

At present, VGGNet [37], Inception [38] and ResNet [39]are popular convolution neural networks. VGGNet [37] investigates the relationship between a convolution neural network’s depth and its performance by using a smaller convolution kernel to introduce nonlinear transformation without affecting input and output dimensions, increasing network expressivity, reducing computation, and training and predicting with the multi-scale method, which can increase the amount of data to be trained, prevent model fitting, and improve prediction accuracy. When VGGNet [37] reaches a certain depth. However, performance saturation occurs. To overcome the aforementioned difficulties, the Inception [38] and ResNet [39] networks were designed. The introduction network is a GoogleNet module that conducts convolutions or pooling operations on incoming photos in parallel and splices all of the outputs into an intense feature map. Within the constraints of computing resources, the network’s performance may be increased further.

By channeling input information directly to the output, a nonlinear modification of inputs before ResNet replacement speeds up neural network training and improves model accuracy and generalization. Although ResNet enables the network to break through hundreds of layers, an intense network might produce issues such as gradient fading and explosions. ResNet and Inception are combined into ResNeXt, created by stacking numerous residuals with the same topology and increasing cardinality [15]. The above networks improve the network’s performance by increasing the width or depth, but these networks need to be manually tuned to achieve a better level. To solve these problems, Google has proposed EfficientNet [40] and EfficientNetv2 [41], which use the compound scaling method, which uses a blending factor *φ* to scale the network’s width, depth, and resolution uniformly. The EfficientNet series networks not only have smaller network parameters but also have higher accuracy. The Fused-MBConv structure is proposed for the slow training time of EfficientNet series networks. The training-aware NAS and scaling are jointly applied to optimize the model accuracy, speed, and parameter size, and the progressive learning method is proposed to reduce the training time.

## Methods

**Fig 1:**
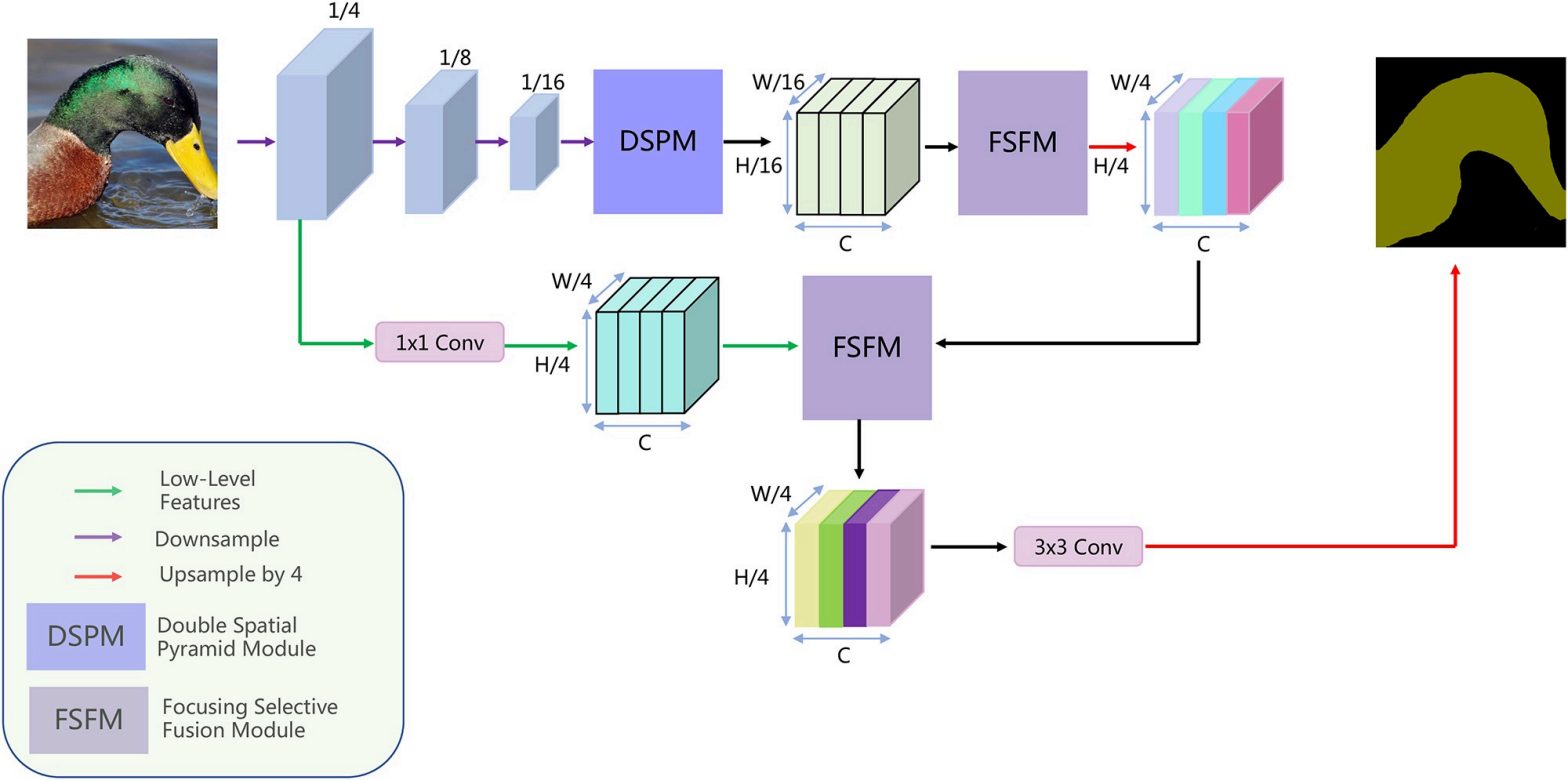
The pipeline of Multi-scale Feature Enhanced Adaptive Fusion Network (MFEAFN).

This section proposes two models: the Double Spatial Pyramid Module (DSPM) and the Focusing Selective Fusion Module (FSFM). DSPM encodes global and multi-scale contextual information. FSFM enhances the adaptive fusion of feature maps at different levels or scales. We propose the Multi-scale Feature Enhanced Adaptive Fusion Network (MFEAFN) based on these two models. Please see Fig 1 for our network structure diagram, which is described in detail below.

### Double spatial pyramid module

**Fig 2:**
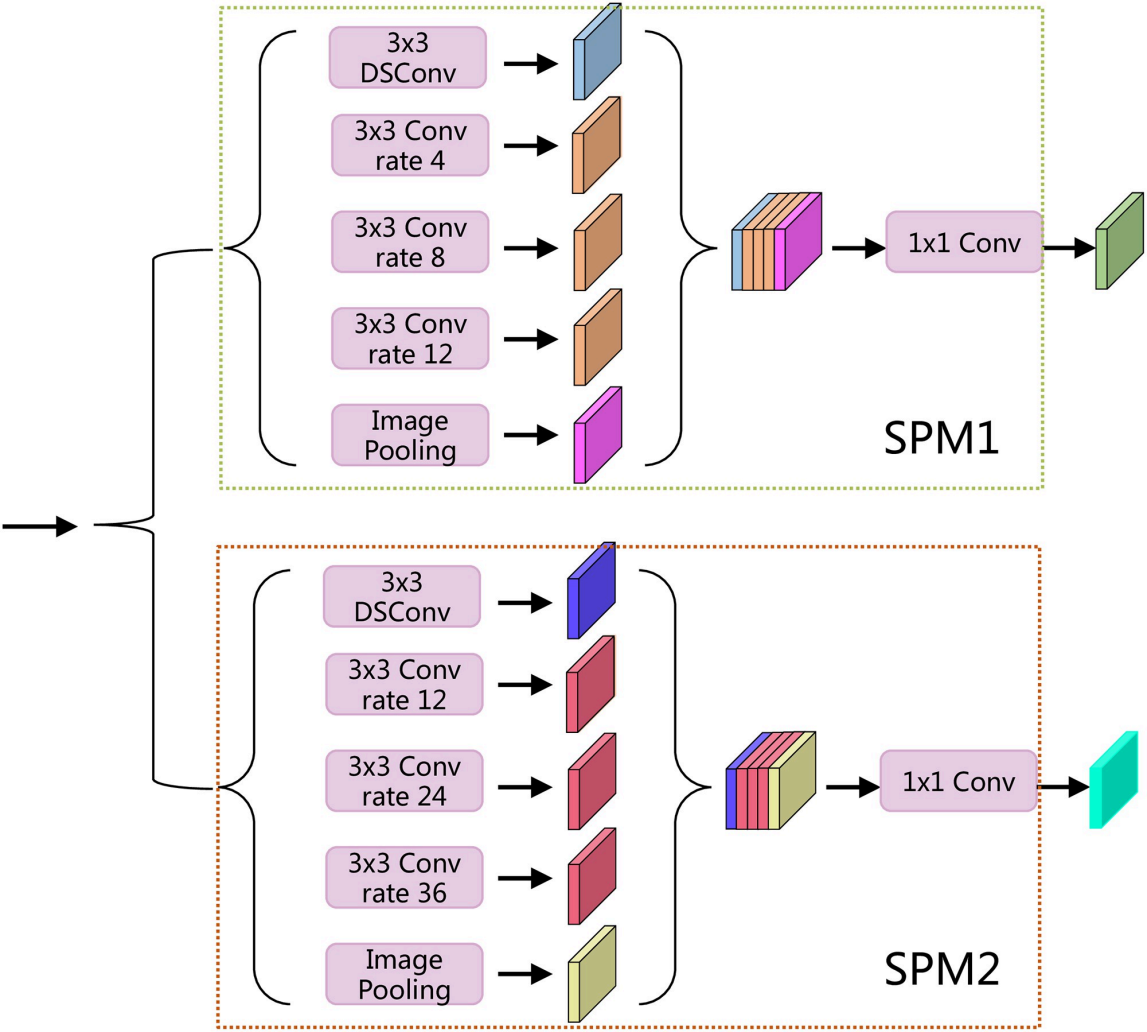
Double Spatial Pyramid Module(DSPM).

For semantic segmentation, context information and global context information are critical. Multiscale context information focuses on aggregating the context information at various scales to aid segmentation for objects of different sizes in the same category. The global information aims to provide a comprehensive understanding of the entire scene by establishing global range dependencies between pixels. Inspired by ASPP [23], we designed the Double Spatial Pyramid Module (DSPM) to obtain global and multiscale context information. The details of the DSPM are shown in Fig 2. For the feature maps $F_{\text{in}}\in R^{H\times W\times C}$ output from the backbone network, we first input them into a two-branch structure composed of two Spatial Pyramid Modules (SPM) in parallel. For SPM1, one 3 × 3 depthwise separable convolution [15] and three 3 × 3 dilated convolutions with different atrous rates are input to capture the multiscale contextual information, and a global averaging pooling operation is used to capture the global information. After that, we use Concatenate operation to stitch the feature map in channel dimension to obtain the feature map $F_{\text{concat}}\in R^{H\times W\times 5C}$, and then use a 1x1 convolution to downscale and interact with the information between channels to obtain the output feature map $F_{\text{out}}\in R^{H\times W\times C}$, and the same for SPM2.

Different receptive fields are required for objects of different sizes that contain the same class in the image. We set the atrous rate r differently in SPM1 and SPM2. In SPM1, the atrous rate r is set to [4,8,12] to capture smaller objects in the image; in SPM2, the atrous rate r is set to [12,24,36] to capture larger objects in the image.

### Focusing selective fusion module

To better fuse the output features of DSPM and different levels of features, we designed three feature fusion methods, as shown in F++ig 4. Ablation experiments demonstrate the optimal performance of the Focusing Selective Fusion Module (FSFM). We focus on the proposed FSFM.

**Fig 3:**
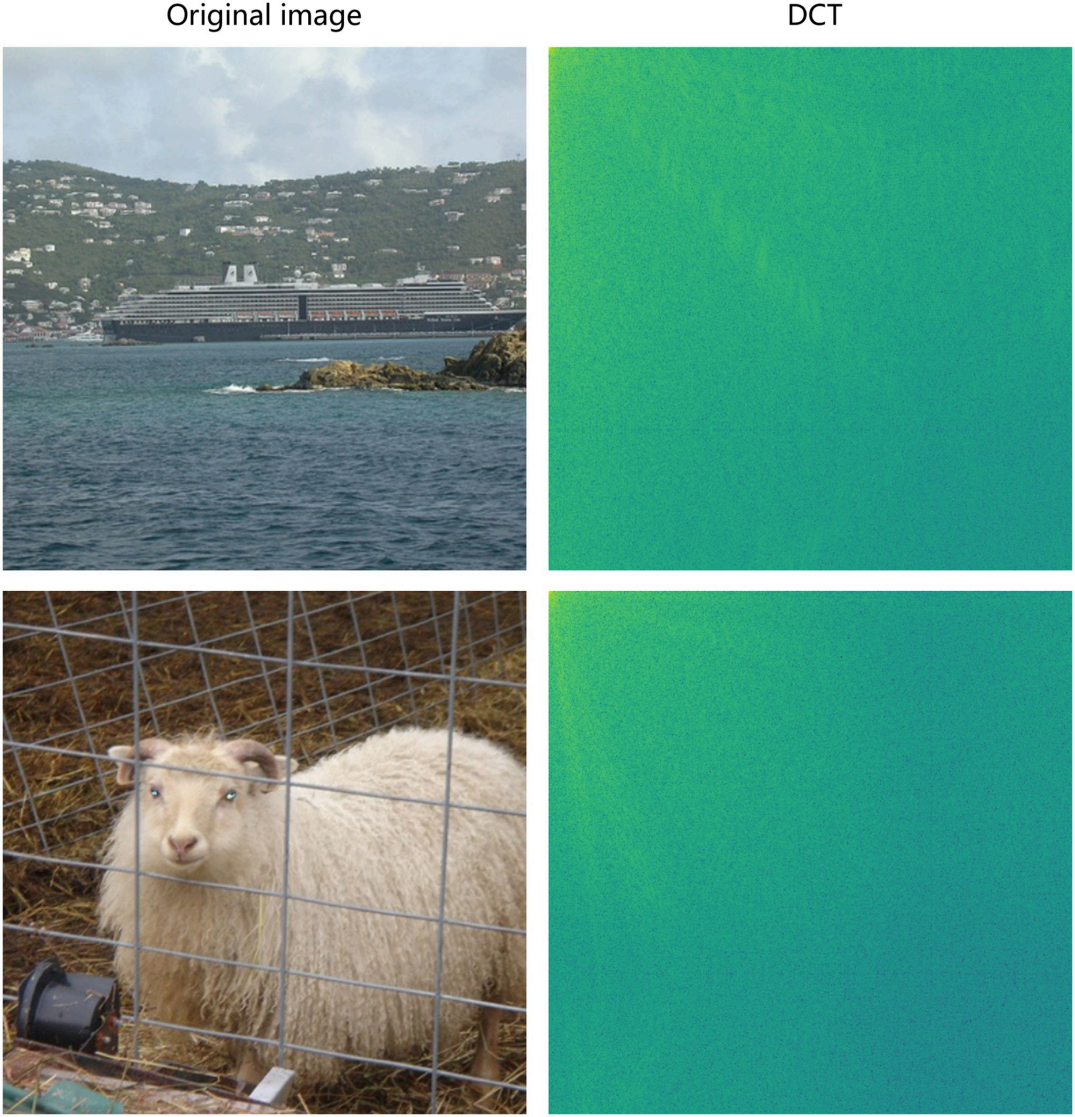
Example of DCT transformation.

Most of the channel attention mechanisms use global average pooling (GAP) operations to obtain a global representation of each channel. However, this approach results in a loss of information details. FcaNet [8] demonstrated that the lowest frequency components of GAP and 2DDCT are proportional. Therefore, we use the two-dimensional discrete cosine transform to obtain the multispectral vector. As shown in Fig 3, the image information is compressed in the upper left corner from two examples of image DCT transformation. Moreover, the channel attention mechanism does not exploit the relationship between different spatial locations. We introduced a spatial attention mechanism based on the designed channel attention mechanism to learn more representative features. In summary, we designed a frequency selective fusion module (FSFM) by using discrete 2DDCT to obtain multiple frequency components for each feature, which then serves as a guide to adaptively assign corresponding weights to feature maps containing relationships between different spatial locations.

The spatial attention mechanism receives feature maps *F*1 and *F*2 and the spatial relationship between them is used to build two two-dimensional spatial weight maps *W*1 and *W*2, which are then multiplied by the appropriate spatial locations to learn more features. We produce feature descriptors by concatenating them using the average pooling and maximum pooling operations. Then, we connect the two feature descriptors using a 7x7 convolution operation to generate the appropriate spatial attention maps. *W*_*s*_ is the spatial attention map computed as follows:

$$\begin{array}{c} W_{s}\left(F\right)=\sigma (f^{7\times 7}\left(\left[\text{Avgpool}\left(F\right);\text{Maxpool}\left(F\right)\right]\right)) \end{array}$$ (1)

The feature maps *F*1 and *F*2 are then fed into the spatial attention mechanism to yield *W*1 and *W*2, respectively, and then multiplied by the corresponding spatial positions to yield the spatial relations feature maps *K*1 and *K*2.

$$\begin{array}{c} K1=W_{1}\left(\;F1\right)⊗F1, \end{array}$$ (2)

$$\begin{array}{c} K2=W_{2}\left(\;F2\right)⊗F2 \end{array}$$ (3)

where ⊗ is element-wise multiplication.

The following, the two feature maps F1 $\in R^{H\times W\times C}$ and F2 $\in R^{H\times W\times C}$ are used to generate the feature map F $\in R^{H\times W\times 2C}$ by the concatenation operation:

$$\begin{array}{c} F=\text{Concat}(F1,\;F2), \end{array}$$ (4)

where *Concat* denotes the concatenate operation.

The feature map *F* is divided into n parts along the channel dimension, [*F*^1^, *F*^2^, ⋯, *F*^*n*^], in which $F^{x}\in R^{1\times H\times W},x\in \left\{1,2,\cdots ,n\right\},n=2C$. The input feature map was split into 2C parts, and each channel in the feature map was converted into a corresponding 2-D DCT. This can compress the multi-frequency component, including the lowest frequency component, to obtain more information. In addition, FcaNet has demonstrated that the lowest frequency components of GAP and 2DDCT are proportional. The forward and inverse transformations of two-dimensional discrete cosine transform are shown in Eq.5 and Eq.6, respectively:

$$\begin{array}{c} D\left(u,v\right)=\alpha \left(u\right)\alpha \left(v\right)\sum_{i=0}^{H-1}\sum_{j=0}^{W-1}f\left(i,j\right)\text{cos}\frac{(2i+1)u\pi }{2H}\text{cos}\frac{(2j+1)v\pi }{2W} \end{array}$$ (5)

$$\begin{array}{c} f\left(i,j\right)=\alpha \left(u\right)\alpha \left(v\right)\sum_{i=0}^{H-1}\sum_{j=0}^{W-1}D\left(u,v\right)\text{cos}\frac{(2i+1)u\pi }{2H}\text{cos}\frac{(2j+1)v\pi }{2W} \end{array}$$ (6)

where $D\left(u,v\right)\in R^{H\times W}$ is the 2-D DCT frequency spectrum, $f\left(i,j\right)\in R^{H\times W}$ is the input feature, *H* and *W* are the height and width of the *f*(*i*, *j*); *u* ∈ {0, 1, ⋯, *H* − 1}, *v* ∈ {0, 1, ⋯, *W* − 1}.

Then, the divided feature maps were substituted into Eq.5 to obtain $D_{freq}^{x}\in R^{1\times 1\times 1},x\in \left\{1,2,\cdots ,n\right\},$ *n* = 2*C*. The whole attention mask vector can be obtained by concatenation operation on the obtained results:

$$\begin{array}{c} D_{freq}=\text{Concat}([D_{freq}^{1},D_{freq}^{2},\cdots ,D_{freq}^{n}]), \end{array}$$ (7)

where $D_{freq}\in R^{2C\times 1\times 1}$ is the obtained mask vector. Then, the mask vectors $D_{freq}\in R^{2C\times 1\times 1}$ through the FRF layer:

$$\begin{array}{c} G=FRF(H_{freq})=W_{4}(W_{3}H_{freq}), \end{array}$$ (8)

where *FRF* layer consists of two 1x1 convolutions $\in R^{C}$ with a convolution kernel size of 5. The *W*3 and *W*4 are parameters of two 1x1 convolutions layers, respectively.

Then, the guide vector G is used to compute attention weights. The guide vectors G were reshaped into two guide tensors P $\in R^{1\times 1\times C}$ and Q $\in R^{1\times 1\times C}$. Inspired by SKNet [6], we adaptively adjust the weight of the input feature maps in the FSFM module. We converted to frequency attention weight vector $W^{A}\in R^{1\times 1\times C}$ and $W^{B}\in R^{1\times 1\times C}$ for *K*1 and *K*2 respectively through the element-wise softmax operation:

$$\begin{array}{c} W_{c}^{A}=\frac{e^{P_{c}Q_{c}}}{e^{P_{c}}+e^{Q_{c}}}, \end{array}$$ (9)

$$\begin{array}{c} W_{c}^{B}=\frac{e^{P_{c}Q_{c}}}{e^{P_{c}}+e^{Q_{c}}}, \end{array}$$ (10)

where $W_{c}^{A}$ is the c-th element of $W^{A}\in R^{1\times 1\times C}$, P_c_ is the c-th element of *P*, likewise $W_{c}^{B}$ and *Q*_*c*_. $w_{c}^{A}+w_{c}^{B}=1$. The fused feature map *N* $\in R^{H\times W\times C}$ is obtained by reallocating attention weights to different convolution kernels:

$$\begin{array}{c} N_{c}=(K1_{c}⊗w_{c}^{A})⊕(K2_{c}⊗w_{c}^{B}). \end{array}$$ (11)

where $N=[N_{1},N_{2},\ldots ,N_{C}],N_{c}\in R^{H\times W}$, *K*1_*c*_ is the c-th row of *K*1, likewise *K*2_*c*_; ⊕ indicates element-wise summation, ⊗ indicates element-wise multiplication.

### Network agriculture

Based on the DSPM and FSFM components, we designed the architecture of the MFEAFN, as shown in Fig 1. Unlike ResNet, which increases width and dimensionality, EfficientNetv2-S uses a hybrid factor *φ* to scale the network’s width, depth, and resolution to improve its performance. Therefore, we employ the EfficientNetv2-S as our backbone. Then, the DSPM is designed to extract both global and multiscale context information from the backbone EfficientNetv2-S by 16 times downsample. Then, we take the output of the DSPM as the input of two FSFM to enhance and adaptively fuse the multiscale context information for objects of different sizes. Previous studies have shown that spatial detail information is important for improving network performance. The low-level features have high resolution and contain rich location and detailed information. The high-level features contain semantic information. The two different levels of features do not contain the same information, so it is impossible to fuse the two features using the concatenate operation. We also use FSFM to enhance the adaptive fusion of these different levels of features. Finally, a 3 × 3 standard convolutional refinement feature is used to obtain the final output after quadruple upsampling.

## Experiments

### The experimental configuration and implementation details and evaluation metrics

The algorithm proposed in our method has been experimentally studied. The experimental software and hardware configuration are shown in Table 1.

**Table 1:** Machine software and hardware configuration.

Project	Detail
CPU	AMD EPYC 7742 64-Core Processor
RAM	32G
Operating System	Ubuntu 18.04.1
GPU	NVIDIA Tesla A100 40G
CUDA	Cuda 11.0
Data processing	Python 3.6

We evaluated MFEAFN with deeplabv3+, mean intersection over union (mIoU), intersection over union (IoU), overall accuracy (OA), and mean pixel accuracy (mPA) on the PASCAL VOC 2012 dataset [42] and the Cityscapes dataset [22] as the evaluation metrics of the model:

$$\begin{array}{c} mIoU=\frac{1}{k+1}\sum_{i=0}^{k}\frac{p_{ii}}{\sum_{j=0}^{k}p_{ij}+\sum_{j=0}^{k}p_{ji}-p_{ii}}, \end{array}$$ (12)

$$\begin{array}{c} IoU=\frac{p_{ii}}{\sum_{j=0}^{k}p_{ij}+\sum_{j=0}^{k}p_{ji}-p_{ii}} \end{array}$$ (13)

$$\begin{array}{c} mPA=\frac{1}{k+1}\sum_{i=0}^{k}\frac{p_{ii}}{\sum_{j=0}^{k}p_{ij}}. \end{array}$$ (14)

where *k* is the classes used in the experiments and *p*_*ii*_, *p*_*ij*_, and *p*_*ji*_ denote the pixel number of true positives, false positives, and false negatives, respectively. IoU measures the ratio of the intersection of a category’s predicted and actual values to their union. mIoU measures the ratio of the intersection of the predicted outcomes and the actual values for each category, summed and averaged. The mPA calculates the proportion of pixels correctly classified for each class separately and then sums and averages them. IoU, mIoU, and mPA are all standard metrics for measuring model performance in semantic segmentation.

### Datasets and implementation details

#### Pascal VOC 2012 and Cityscapes

Original PASCAL VOC 2012 dataset [43] contains 1, 464 (train), 1, 449 (Val), and 1, 456 (test) pixel-level annotated images and 20 foreground object classes, and one background class. We augment the dataset with the extra annotations provided by [42], resulting in 10, 582 (trainaug) training images. In the experiments, In our experiments, we use the “poly” strategy [44] as our learning rate strategy, with the initial learning rate set to 0.01, weight decay to 0.0005, SGD network model optimizer, the momentum of 0.9, batch size 16, crop size 512 512, and 50 epochs.

The Cityscapes dataset contains street scenes from 50 different cities, in addition to high-quality annotations of 5000 pixel-level frames (2975, 500, and 1525 for the training, validation, and test sets, respectively) and 20,000 coarsely labelled images. The experiments’ initial learning rate is set to 0.1, batch size to 8, crop size of 768 × 768, and 80 epochs.

### Ablation study on PASCAL VOC2012 val set

#### Ablation study of DSPM on PASCAL VOC2012 val set

In Table 2, the first line uses the ASPP from the original paper [23], and the second line uses an additional 3 × 3 dilated convolution with atrous rates of 24 for obtaining long-range dependencies in the ASPP module. Experimental results show that the network’s performance degrades by 0.11% compared to the original ASPP. To further improve the network’s performance, we tried to replace the 1 × 1 convolutional layer of ASPP with a 3 × 3 depth-separable convolutional layer. As seen from the first and fourth lines of Table 2, MASPP slightly improves compared with ASPP. As seen from the first and fourth lines of Table 2, MASPP slightly improves compared with ASPP.

**Table 2:** Ablation results on the PASCAL VOC 2012 valuation set. ASPP is Atrous spatial pyramid pooling. MASPP refers to replacing the 1 × 1 convolution layer of ASPP with a 3 × 3 depthwise separable convolution layer. DSPM is our Double Attention Pyramid Module.

Method	Atrous Rate	mIoU(%)	batchsize
ResNet50+ASPP [23]	(6,12,18)	75.60	16
ResNet50+ASPP [23]	(6,12,18,24)	75.49	16
ResNet50+PPM [2]	-	76.16	16
ResNet50+MASPP	(6,12,18)	75.82	16
ResNet50+DSPM	(4,8,12),(12,24,36)	76.79	16

#### Ablation study of FSFM on PASCAL VOC2012 val set

**Fig 4:**
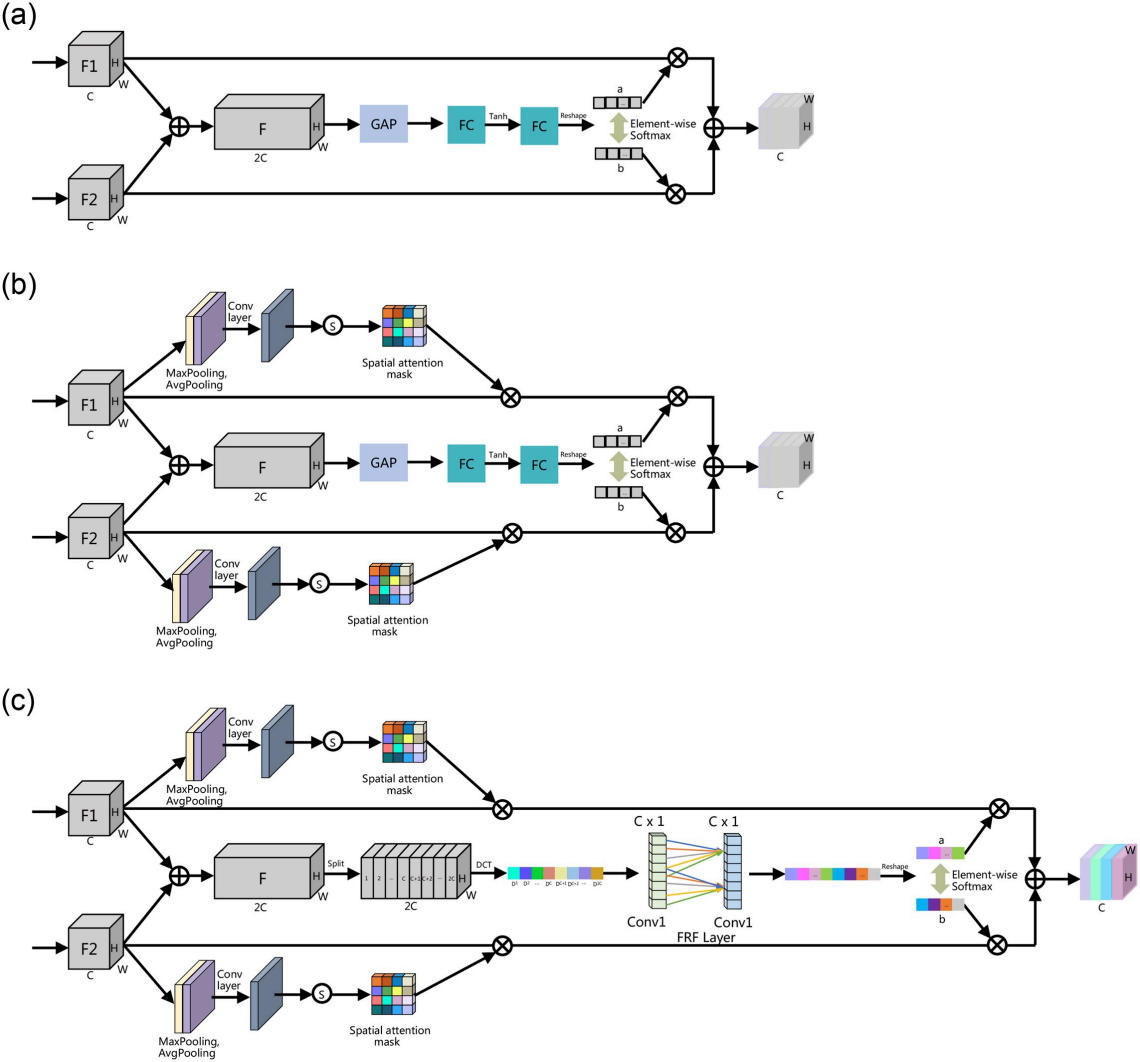
Three different feature fusion modules.

To verify the effectiveness of the proposed feature fusion module FSFM, we conducted ablation experiments as shown in Table 3. We used ResNet101 and DSPM as the baseline. We compare the four feature fusion modules in Fig 4. As shown in Table 3, using the commonly used concatenation is 0.58% mIoU higher than the baseline network, which proves that the Concat method of fusing features can effectively improve the performance of the network. Compared with line 3 with line 2 of Table 3, we replace the concatenation operation with SFM, and the mIoU acquires a further improvement from 79.08% to 79.35%, which proves the effectiveness of the SFM. Because SFM can adaptively fuse the required features at different scales, From line 3 to line 4, we observe that we add the spatial attention mechanism module SSFM to both branches of SFM separately, which is 0.3% mIoU higher than SFM. Because SSFM not only adaptively selects essential information in the channel dimension but also emphasizes or suppresses information in the spatial dimension. The FSFM designed by replacing the global average pooling (GAP) with 2DDCT on top of SSFM obtains 80.36% mIoU and the best network performance compared with the above three feature fusion methods. Because 2DDCT can obtain more information, including GAP in the adaptive selection of the focused information through the attention mechanism.

**Table 3:** Ablation results on the PASCAL VOC 2012 valuation set. Concat refers to the use of concatenation operations to fuse the output of DAPM. SFM: Selective Fuse Module. SSFM is the addition of the Spatial Attention Mechanism to each of the two branches of SFM. FSFM refers to the Focusing Selective Fuse Module.

Class	Baseline	Concat	SFM	SSFM	FSFM
background	93.55	94.61	94.03	94.16	94.05
aeroplane	90.36	92.76	92.16	92.48	92.13
bike	41.12	41.55	41.78	42.34	42.82
bird	91.78	90.67	90.84	91.35	92.14
boat	71.14	74.74	74.34	74.85	75.98
bottle	81.19	82.18	81.69	81.19	82.16
bus	95.20	94.05	95.24	95.51	95.52
car	88.49	87.63	87.97	88.86	90.87
cat	94.19	94.36	94.12	94.23	93.89
chair	36.50	39.74	42.08	42.17	43.27
cow	87.17	88.29	89.57	90.64	91.09
table	60.19	59.02	56.53	56.79	56.84
dog	90.56	89.88	90.82	91.63	92.10
horse	88.26	86.73	87.65	87.23	90.26
motorbike	86.30	86.39	87.73	87.48	88.38
person	85.33	87.38	86.35	86.05	86.97
plant	67.40	62.10	66.68	69.09	69.69
sheep	83.61	89.62	88.64	88.76	87.61
sofa	51.96	52.89	53.01	53.21	53.97
train	88.12	88.39	88.57	89.12	90.85
monitor	76.06	77.83	77.67	77.56	77.94
mIoU	78.50	79.08	79.35	79.65	80.36
OA	94.52	94.71	94.74	94.76	94.84
mPA	88.02	88.39	88.63	88.95	89.34

**Fig 5:**
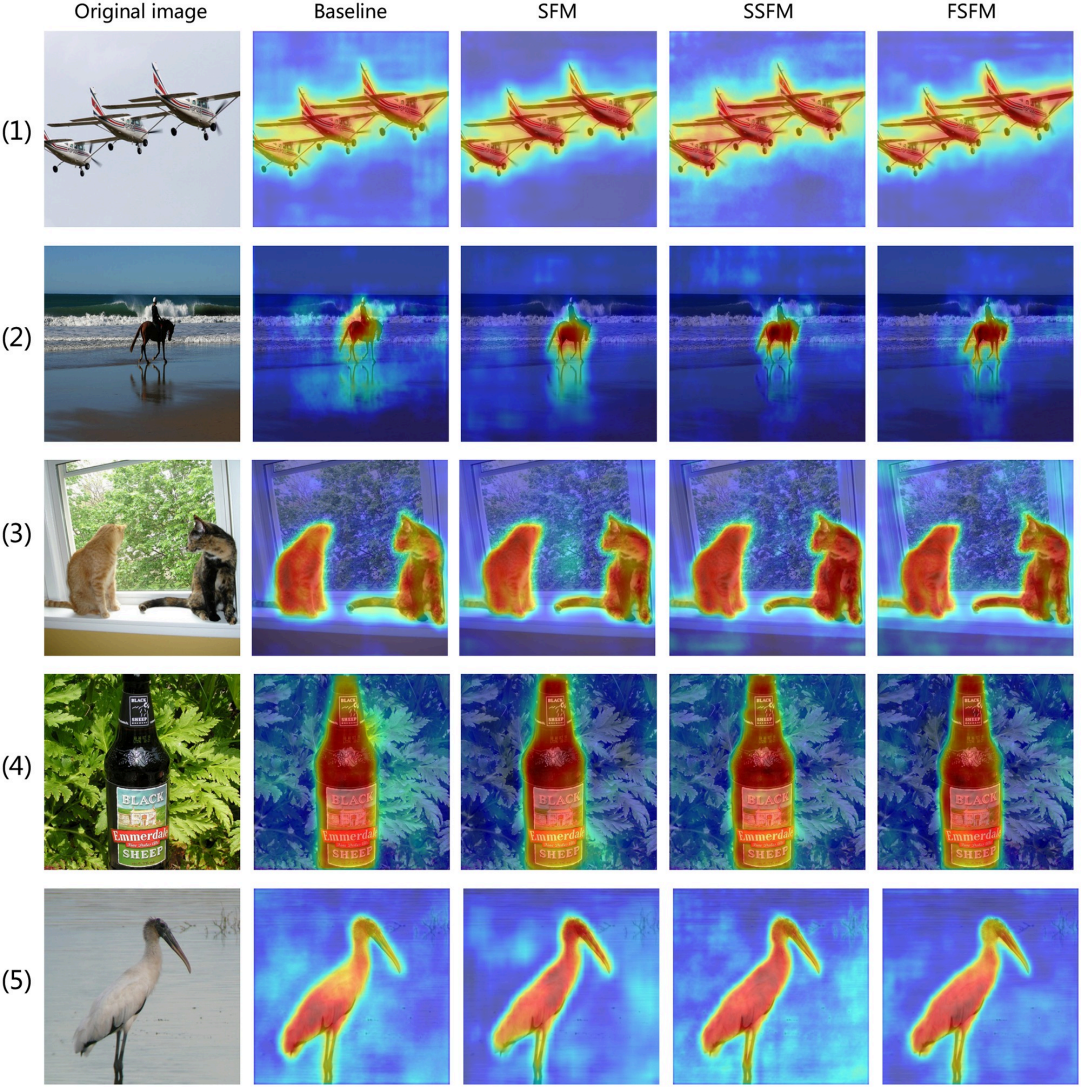
Grad-CAM [45] visualization result and the Grad-CAM visualization are calculated for the last convolutional outputs. The first column is the original image. We compare the visualization results of the proposed three different feature fusion modules with the baseline.

To get a more intuitive understanding of the function of our proposed feature fusion module, we visualized some images from the PASCAL VOC2012 dataset, as shown in Fig 5. We can see that our method not only focuses well on single or multiple objects containing only one category of objects in the picture but also on different categories of objects visually.

### Comparison with state-of-the-art methods

#### Results on PASCAL VOC2012 set

**Fig 6:**
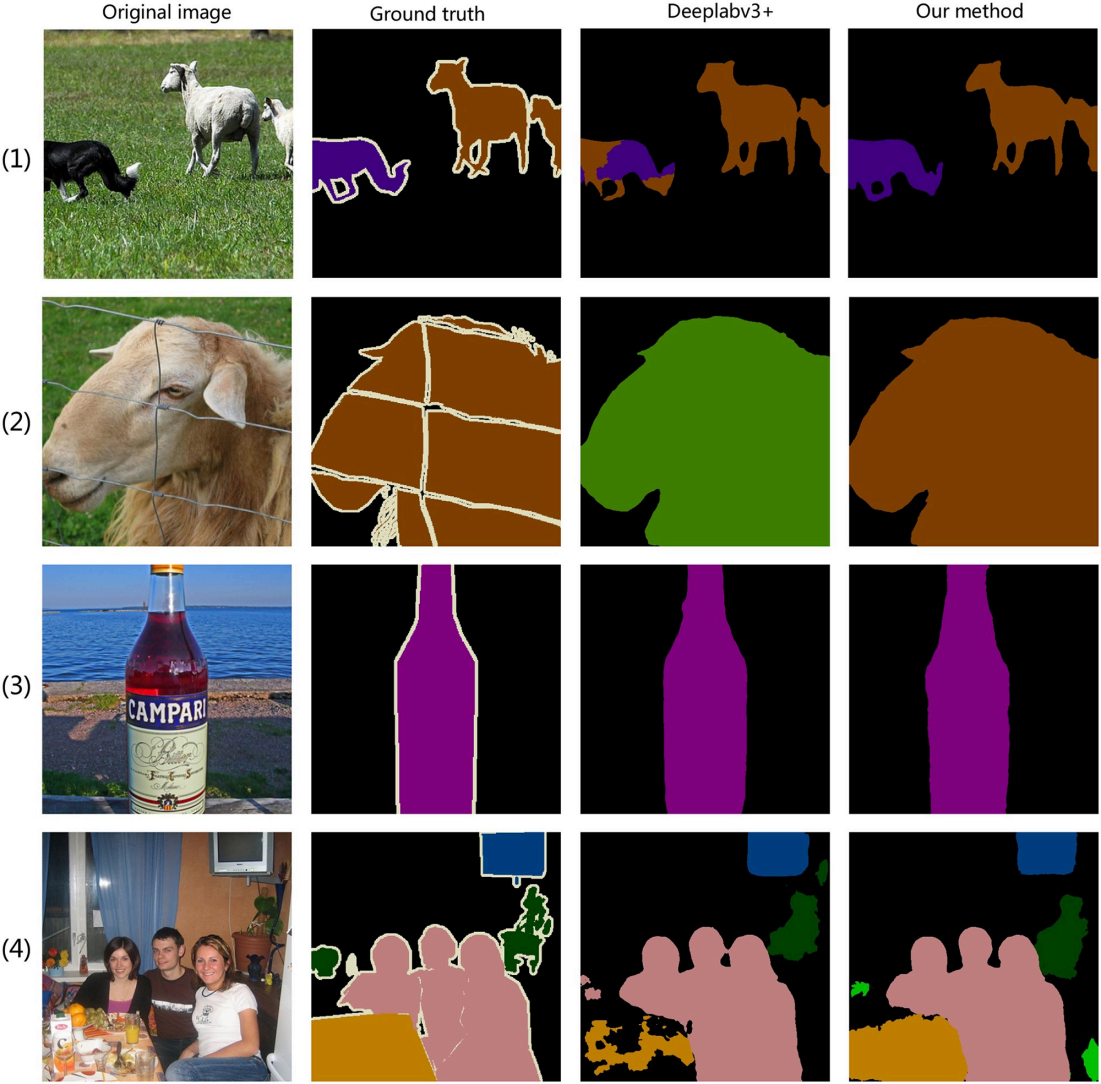
Comparison of DeepLabv3+ and MFEAFN visualization results on PASCAL VOC 2012 validation set.

We compare DeepLabv3+ with MFEAFN to further verify the validity of the proposed MFEAFN. Four visual examples of the segmentation results of MFEAFN and DeepLabv3+ on the PASCAL VOC2012 validation set are shown in Fig 6. The first and second columns show the original and labelled images, respectively. The third column shows the segmentation results of DeepLabv3, while the fourth and fifth columns represent the segmentation results when MFEAFN employs ResNeXt 101 and EfficientNetV2 as the backbone networks, respectively. For the example image in the first row of Fig 6, DeepLabv3+ incorrectly classifies the pixels in the most detailed regions of the “dog” as “sheep”, i.e., the front half of the dog’s body, limbs, and part of the tail. Due to the similar object structure, DeepLabv3+ incorrectly classifies “sheep” as “cattle” in the second row of Fig 6, thus causing class confusion. In the third row of Fig 6, MFEAFN segmented the “bottle” object with smoother edge contours than DeepLabv3+. In the fourth row of Fig 6, MFEAFN segmented the “table” object more entirely and with more apparent figure contours than DeepLabv3+. These results show that MFEAFN better segmented detailed areas and similar objects. The fifth column in Fig 6 is more precise than the fourth in terms of the detail of the divisions; for example, the complete edge detail of the divisions “sheep,” and “cow,” and the complete outline of the “bottle.”.

**Fig 7:**
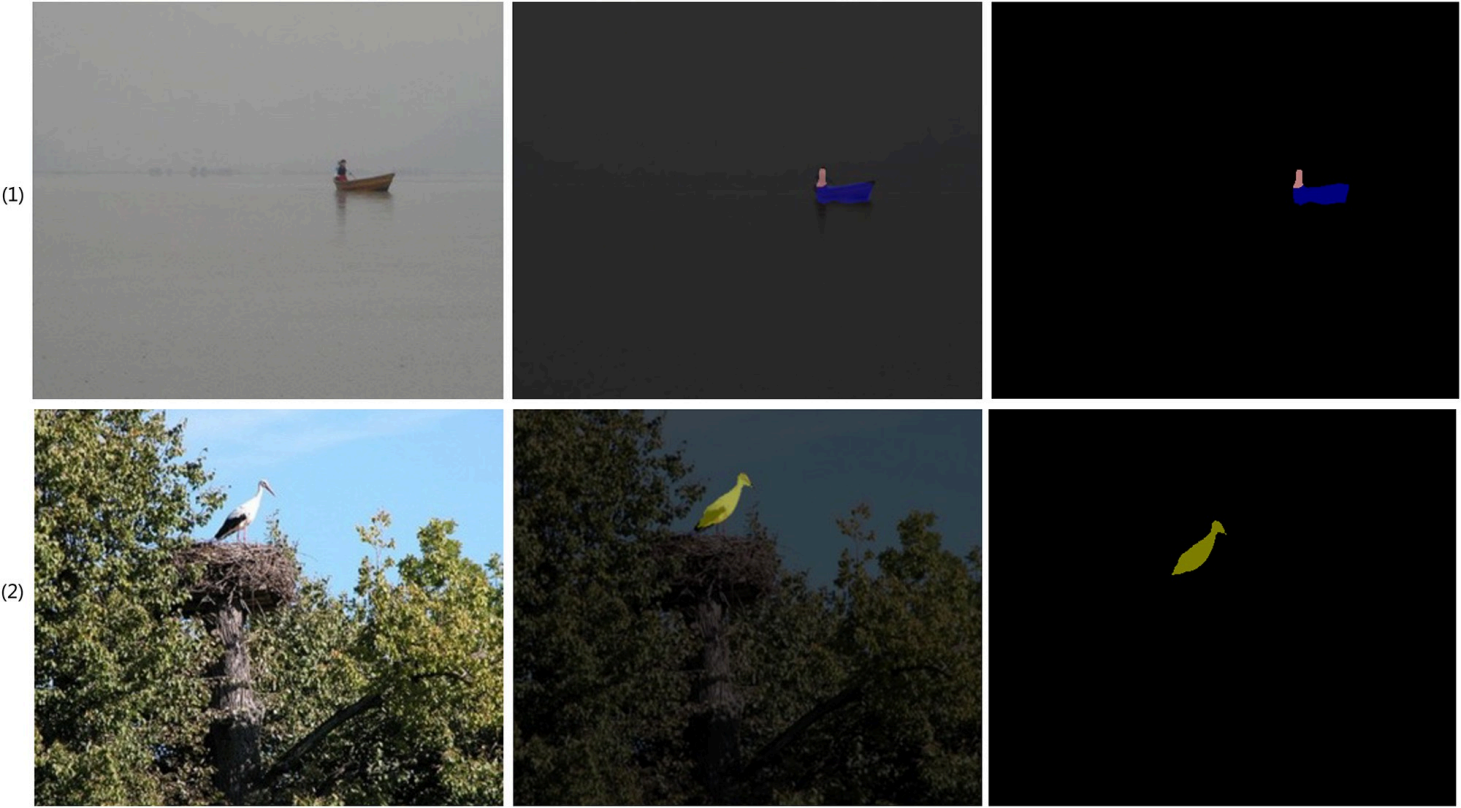
Some examples of MFEAFN failures’ visualisation results on the the PASCAL VOC 2012 validation set.

Some examples of MFEAFN failures are given in Fig 7. The original image is shown in the first column, and the output module of DeepLabv3+ and the output module superimposed on the original image are shown in the second column. The third column shows the predicted map of the MFEAFN output. The second column in Fig 7 shows that the MFEAFN mask does not cover the original image well for small distant objects such as “boats,” and “birds,” and that there is room for further improvement of our proposed method for small foreign objects.

In addition, we compared the MFEAFN proposed in this paper with other segmentation networks on the PASCAL VOC2012 validation dataset. The comparison results are shown in Table 4.

**Table 4:** Comparison with state-of-the-art Meth on PASCAL VOC 2012 set.

method	year	MIoU(%)
FCN [1]	2015	62.20
DPN [12]	2015	74.10
DeepLabv2 [23]	2017	71.60
DeepLabv3 [3]	2017	78.50
Deeplab-CRF [23]	2017	77.69
PSPNet [2]	2017	79.63
DeepLabv3+ [18]	2018	78.85
DANet [30]	2019	80.40
APCNet [25]	2019	80.71
EMANet [33]	2019	81.32
DSNet [24]	2020	81.63
SpineNet-Seg [26]	2021	81.40
MFEAFN (ResNeXt101)	-	81.32
MFEAFN (EfficientNetv2-S)	-	82.64

#### Results on Cityscape set

**Fig 8:**
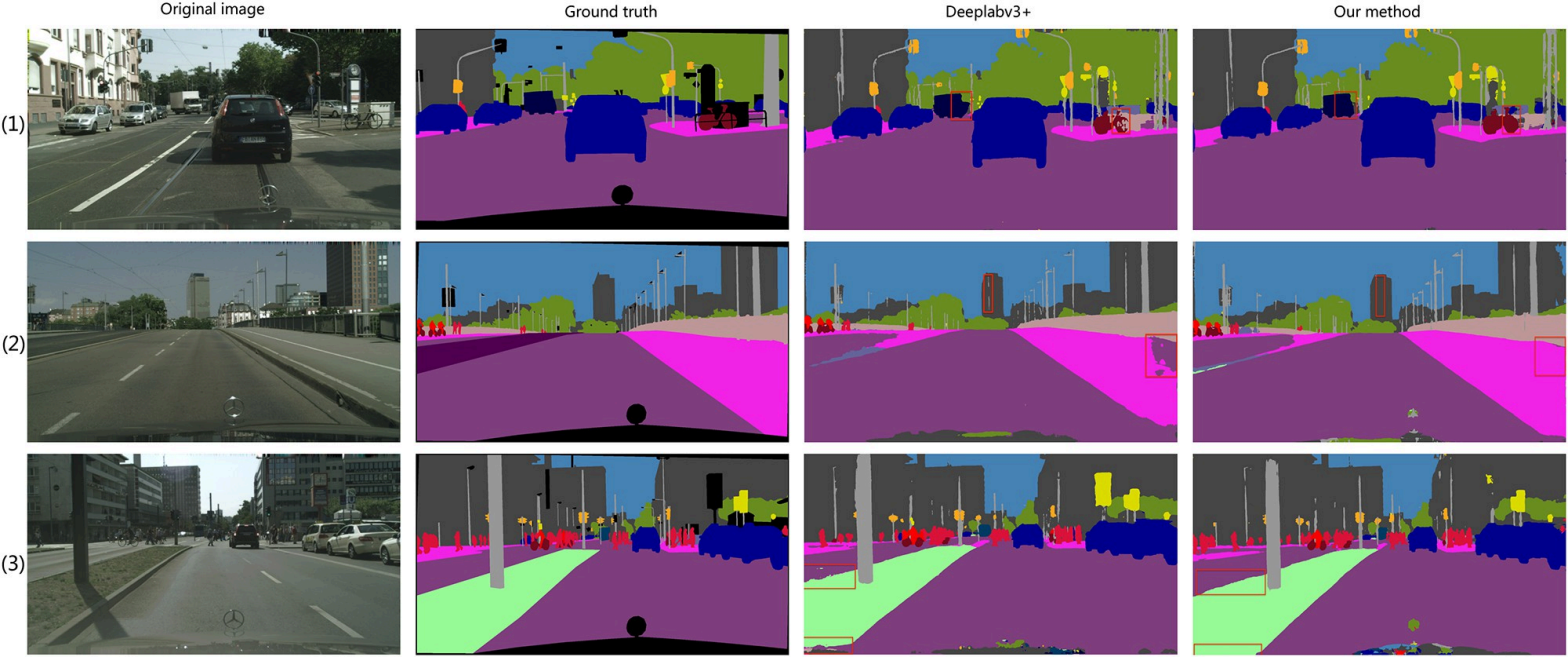
Comparison of DeepLabv3+ and MFEAFN visualization results on Cityscapes validation set.

We further examine the generalization ability of the MFEAFN model on the Cityscapes dataset. Four visual examples of the segmentation results of MFEAFN and DeepLabv3+ on the Cityscapes dataset are shown in Fig 8. The first and second columns show the original and labelled images, respectively. The third column shows the segmentation results of DeepLabv3, while the fourth and fifth columns represent the segmentation results when MFEAFN employs ResNeXt 101 and EfficientNetV2 as the backbone networks, respectively. For the example image in the first row of Fig 8, the MFEAFN segmentation of the bicycle in the image is more complete than the Deeplabv3+ network, and the boundary of the car is predicted more carefully. In the second row of Fig 8, DeepLabv3+ incorrectly classifies the pixels in the detail area of the “building” as “pole”. It causes the segmentation effect to lose detailed information on the “sidewalk” in the lower right corner. In line 3 of Fig 8, DeepLabv3+ incorrectly predicts the “terrain” category to the “sidewalk” category, causing class confusion and missing edge details. The fifth column in Fig 7 is more continuous in its segmentation of objects than the fourth; for example, the segmentation of “traffic light” and “pole” is more contiguous.

In addition, we also compare the MFEAFN proposed in this paper with other segmentation networks on the Cityscapes validation dataset. The comparison results are shown in Table 5.

**Table 5:** Comparison with compared with state-of-the-Art on the Cityscapes dataset.

method	year	Parameters(M)	FLOPs(G)	MIoU(%)
FCN-8S [1]	2015	11.80	37.45	62.20
DPN [12]	2015	-	-	74.10
ICNet [22]	2016	7.8	-	9.50
DeepLabv1 [23]	2017	262.1	457.8	63.10
DeepLabv3 [3]	2017	-	-	75.23
DeepLabv3+ [18]	2018	-	-	76.28
DFANet B [46]	2019	4.8	2.1	67.10
DFANet A [46]	2019	7.8	3.4	71.30
BiSeNetV2 [47]	2021	156.0	21.2	72.60
BiSeNetV2-L [47]	2021	47.3	118.5	75.30
CFPNet-V1 [27]	2021	0.31	-	60.40
CFPNet-V2 [27]	2021	0.37	-	66.50
CFPNet-V3 [27]	2021	0.55	-	70.10
HyperSeg-M [48]	2021	10.1	7.5	76.20
HyperSeg-S [48]	2021	10.2	17.0	78.20
FPANet A [28]	2022	14.11	69.21	72.00
FPANet B [28]	2022	-	-	73.70
FPANet C [28]	2022	15.45	88.11	75.90
MFEAFN (EfficientNetv2-S)	-	24.61	43.72	78.46

## Conclusion

We designed a Double Spatial Pyramid Module (DSPM)to extract objects of different sizes in the same category more efficiently. In addition, to better fuse the characteristics of different scales or levels, we built the Frequency Selective Fusion Module (FSFM), which can enhance the adaptive fusion of these features by generating spatial and frequency correlation weight mappings for each feature map. Based on the DSPM and FSFM Module, we propose a multi-scale feature enhancement adaptive fusion network (MFEAFN) that effectively solves the problems of local information loss and class confusion. Experimental results of the proposed algorithm on the PASCAL VOC 2012 and Cityscapes data sets show that MFEAFN has better segmentation performance than state-of-the-art methods.
